# Medical Witnesses Shall Receive Compensation as Experts

**Published:** 1878-04

**Authors:** James P. White

**Affiliations:** Buffalo


Buffalo, March 18, 1878.

MESSRS. EDITORS.

By inserting in your valuable JOURNAL the enclosed extract from the *Fort Wayne Daily Sentinel*, you, I doubt not, will confer a great favor upon all your readers who are liable to be called upon to testify as experts.

Respectfully Yours, &c.

JAMES P. WHITE.

### MEDICAL WITNESSES SHALL BE PAID AS EXPERTS.

The supreme court of the State of Indianna has decided that medical witnesses, when they give their opinions merely, shall be compensated therefor. Below will be found the opinion in full.

"6,764. Alpheus P. Buchman vs. the State of Indiana: Allen C. C. Reversed.

Worden, J.—On the trial of one Hamilton, on a criminal charge, in the Allen criminal court, Dr. A. P. Buchman was put upon the stand as a witness. After stating his profession, the length of time he had been engaged in the practice of medicine, and answering the other usual questions in regard to residence, etc., he was asked to give his professional opinion as a medical expert in regard to certain questions raised in the case. He replied: "The answer that I would have to give would depend on my professional knowledge of the subject, and I respectfully refuse to give my professional opinion without being compensated." The court below being of opinion that the witness was required by law to answer the questions without compensation other than ordinary witness fees, and the witness persisting in his refusal to answer, he was committed for contempt. From the commitment the witness appeals to this court.

The question presented is a novel and important one in this state. It must be conceded that a physician or surgeon, when called upon, must attend and testify to facts within his knowledge; he stands upon an equality in reference to compensation with all other witnesses. But the question is whether he can be compelled to give a professional opinion without compensation other than the ordinary fees to witnesses.

The learned judge reviews at great length and exhaustively, all the English and American cases bearing directly or analogously upon the question raised in the case, and holds:

In respect to facts, the law and his duty to the public require him to attend and testify for such fees as the legislature have provided. Not so, however, in respect to his professional opinions. In giving them he is performing a "particular" service, which cannot be demanded of him without compensation. The thirteenth section of the bill of rights provides that in all criminal prosecutions the accused shall have compulsory process for obtaining witnesses in his favor. The term witnesses, as thus used, was used in its ordinary sense, embracing those who know, or are supposed to know, some fact or facts pertinent to the case. But the physician or surgeon, when giving his professional opinion in court, does not occupy the position of a witness testifying to facts. He performs the service under oath to be sure, and this is the only circumstance from which he can be called a witness at all. So the judge upon the bench, the lawyer at the bar, and the jury in the box, all perform their services under oath. It is unnecessary to determine in this case whether all classes of experts can require payment before giving their opinions as such. It is sufficient to say that physicians and surgeons, whose opinions are valuable to them, as the source of their income and livelihood, cannot be compelled to perform service by giving such opinion in a court of justice without such payment. The commitment of appellant for contempt is erroneous. Judgment reversed.

Biddle, C. J. and Niblack, J. dissenting.

6,758. Thomas J. Dills vs. the State of Indiana. Allen C. C. Reversed.

Biddle, C. J.—The case presents the same question as decided in Buchman vs. the State, and on the authority of that case is reversed. Biddle, C. J and Niblack, J. dissenting."

